# Genetic diversity and population structure of a mini-core subset from the world cowpea (*Vigna unguiculata* (L.) Walp.) germplasm collection

**DOI:** 10.1038/s41598-018-34555-9

**Published:** 2018-10-30

**Authors:** Christian Fatokun, Gezahegn Girma, Michael Abberton, Melaku Gedil, Nnanna Unachukwu, Olaniyi Oyatomi, Muyideen Yusuf, Ismail Rabbi, Ousmane Boukar

**Affiliations:** 1 International Institute of Tropical Agriculture (IITA), Ibadan, Nigeria; 2 International Institute of Tropical Agriculture (IITA), Kano, Nigeria; 3 Botany and Plant Pathology Department, Purdue University, West Lafayette, IN 47907-2054 USA

## Abstract

The International Institute of Tropical Agriculture maintains the world’s largest collection of cowpea germplasm of over 15,000 accessions. A sub-set of 298 lines from the loosely composed mini core collection of 370 landraces were genotyped based on genotyping by sequencing (GBS). Ward’s minimum variance hierarchical cluster analysis, model-based ancestry analysis and discriminant analysis of principal component (DAPC) were carried out on this sub-set. Three clusters were identified by the different clustering methods. Principal component analysis further supported the three clusters especially when accessions are scattered along the axes of the first two principal components. The first two principal components explained a total of 22.30% of the variation. Cluster one comprises 115 accessions from the largest number of countries and has the highest gene diversity, heterozygosity and polymorphic information content (PIC) values. Cluster two is made up of 102 accessions, 90 percent of which are from West and Central Africa. Analysis of molecular variance shows that the most variation is among accessions and lowest among clusters. No cluster is made exclusively of accessions from a single country. Based on SNP markers, the sub set of cowpea mini core germplasm collection used in this study encompasses the diversity in the crop.

## Introduction

Cowpea is one of the important grain legume crops in sub-Saharan Africa (SSA) where it contributes to food and nutritional security, fodder for livestock, soil fertility following N fixation as well as income for farmers and food vendors. It is native to SSA where the greatest genetic diversity for this crop exists. While the centre of diversity of cowpea’s wild progenitor is suggested to be in eastern and southern Africa, based on the presence in the sub-region of several primitive, weedy and wild types^1,2^, that of the cultivated type is in West Africa^3^. More than 75% of the over 15,000 accessions of cultivated cowpea germplasm collected from about 90 countries^4^ and maintained at the International Institute of Tropical Agriculture (IITA) are from the West Africa sub-region^5^. Arguably, this supports the assertion that the greatest diversity exists in the sub-region which therefore confirms the area as the crop’s center of origin^6^. Besides IITA, the United States Department of Agriculture – Genetic Resources Information Network (USDA-GRIN) at Griffin USA^7^ and University of California, Riverside, USA^5^ are also conserving collections of about 7,737 and 6,000 cowpea germplasm accessions respectively. Sustained progress in the development of new improved varieties with desirable attributes in any crop depends heavily on the available germplasm, hence the need for their collection and conservation. Such collections are sources of genes needed for enhancing productivity of new, improved varieties in the short, medium and long terms. Due to the often large number of accessions in gene banks, many of them are typically not utilized for crop improvement. To overcome this challenge, the concepts of core^8^ and mini core collections^9^ were proposed in which about ten- and one- percent of the total germplasm collections respectively, are selected to be representatives of the crop’s genetic diversity. The smaller number in the core or mini core collections makes characterization and exploitation of the germplasm for crop improvement more realistic^10^.

Some studies aimed at understanding the genetic diversity in cowpea using molecular markers have been reported. Restriction fragment length polymorphism (RFLP) markers were used to study genetic variation in the genus *Vigna* which comprised cowpea accessions and some of its wild relatives^11^. The RFLP markers separated accessions of cultivated cowpea from the wild relatives as well as from the Asiatic *Vigna* species. Gene derived markers and sequencing were successful in revealing more polymorphisms among *Vigna* species than within species^12^. Genetic variation among cowpea breeding lines was evaluated using simple sequence repeats (SSR) markers and a high level of homozygosity detected^13^. The study also revealed that some recent breeding lines derived from crosses involving several unimproved germplasm lines showed relatively higher levels of genetic diversity. One thousand two hundred (1,200) single nucleotide polymorphism (SNP) markers were used to analyse genetic variation among 422 cowpea accessions and the study revealed the existence of two cowpea genepools, one for West Africa and the second for East Africa^5^. These authors also reported that genetic variation among landraces from outside of Africa was slightly higher than within African landraces.

Next-Generation Sequencing (NGS) based genotyping procedures such as Genotyping-by-Sequencing (GBS) represent a high-marker density approach compared with previous technologies such as RFLP, amplified fragment length polymorphism (AFLP) and SSR. GBS is based on reducing genome complexity with restriction enzymes, coupled with multiplex NGS for high-density SNP markers discovery^14^. The genome-wide molecular marker discovery, highly multiplexed genotyping, flexibility and relatively low cost make GBS an excellent tool in plant genetics and breeding^15,16^. GBS is increasingly being used in both genetic diversity analyses of cultivated and wild crop relatives^17,18^, development of genetic linkage maps^19^, genomic selection and genome wide association studies^20,21^ GBS was used to study genetic diversity among 768 cowpea germplasm lines and the results indicated that the route of the migration of cowpea germplasm to different parts of the globe could be traced to the two candidate original areas of West and East Africa^7^.

Despite their usefulness in studying genetic diversity in crop species, different types of molecular markers are not equally informative. For example, SSR markers were found to be more informative in the classification of some wild rice lines when compared to SNP even though a higher number of the latter marker type was detected^22^. On the other hand, it was reported that in grape the core lacked a quarter of alleles found in the whole *sativa* collections when SSR markers were used^23^. However, each of the three grape core collections contained almost the whole diversity of the crop when 704 SNP markers were utilized. This suggests that there would be limitations in interpreting results of genetic diversity in grape based on SSR. In addition, many of the marker systems are constrained by high costs required for large-scale analyses^24^. The allele specific SNP markers have limitations as they require upfront investments including time and cost for discovery of informative SNPs and assay development. However, the high throughput genome complexity reducing procedures such as GBS have advantages over the conventional and allele specific SNP markers as they enable simultaneous discovery and genotyping of genome wide SNP markers within a shorter time and at lower cost.

As much as molecular markers can help in understanding the extent of genetic diversity in crop germplasm and its management, caution need be exercised in applying results obtained from molecular marker-based diversity studies especially as they pertain to germplasm conservation efforts. This is because in the process of evolution, adaptiveness plays a major role in the survival of individuals in populations and it is not easy to determine if plant selection acts on markers directly or on associated loci responsible for adaptive properties. Raybould *et al*.^25^ reported that molecular markers detected significant gene flow in the United Kingdom’s *Beta maritima* populations whereas isozymes analyses suggested very little gene flow. The authors explained this observation as evidence of selection for traits associated with isozymes. A study of closely related *Avena barbata* and *A. hirtula* and the analysis of multi-locus dynamics through several generations support that inbreeding, even when coupled with tight linkage does not necessarily hold the genotypes together^26^. However, if the adaptive properties are due to associated loci, they behave as a virtually indivisible unity with the genetic markers analysed. The development and maintenance of plant’s adaptiveness within populations are favoured by self-pollination which also enhances the development of spatial differentiation^27^. It is advisable, therefore, that when making germplasm collections for conservation purposes, efforts should focus not only on alleles that contribute to adaptiveness but also multi-locus combinations of alleles that interact with one another in an epistatic favourable way^26^. In the current study, we carried out GBS on 298 of the cowpea mini core lines to understand the underlying genetic diversity and population structure among the cowpea germplasm assemblage maintained at IITA.

## Results

### Genetic diversity

The 298 lines used in this study were collected from 50 countries and they make up most (about 81%) of the cowpea mini core (list of mini core lines used in Supplementary Table 1). The number of lines from Nigeria (55) is the highest among the mini core sub-set followed by those from India (33) and Republic of Niger (24). However, among the 370 mini core lines there are 72, 37 and 33 from each of the three countries representing 19.4%, 10.0% and 8.9% of accessions respectively.

**Figure 1:**
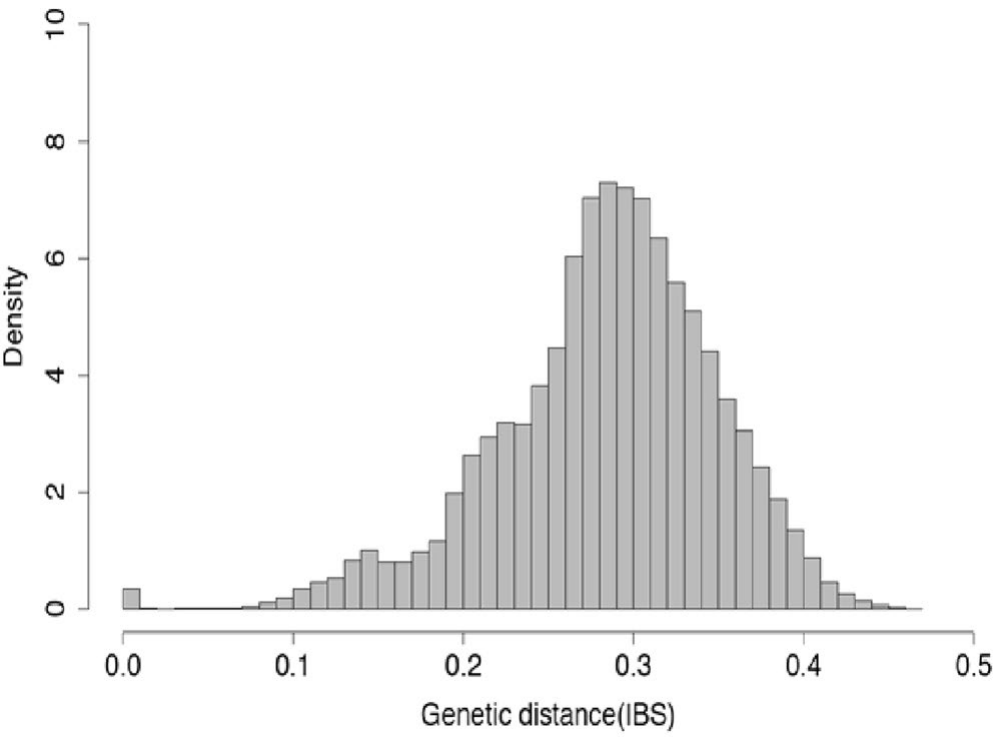
Genetic distance based on identity by state (IBS) among cowpea accessions.

The genetic distance based on differences at marker loci between pairs of the selected accessions range between 0.0096 and 0.462 (Fig. 1). Diversity indices statistics indicate average minor allele frequency (MAF) of 0.209 and polymorphic information content (PIC) of 0.234 while inbreeding coefficient vary widely from as low as −0.438 to as high as 0.929 with a mean of 0.746. The mean expected heterozygosity (0.296) is higher than observed heterozygosity (0.075) values (Table 1).

**Table 1:** Diversity indices statistics based on 2,276 SNP markers and 298 cowpea accessions.

	Ho	He	MAF	PIC	Fis
Min	0	0.095	0.05	0.09	−0.438
Median	0.038	0.175	0.097	0.248	0.834
Max	0.989	0.50	0.50	0.375	0.929
Mean	0.075	0.296	0.209	0.234	0.746

Ho = observed heterozygosity, He = expected heterozygosity, MAF = minor allele frequency, PIC = polymorphic information content, F_IS_ = inbreeding coefficient.

### Population structure

**Figure 2:**
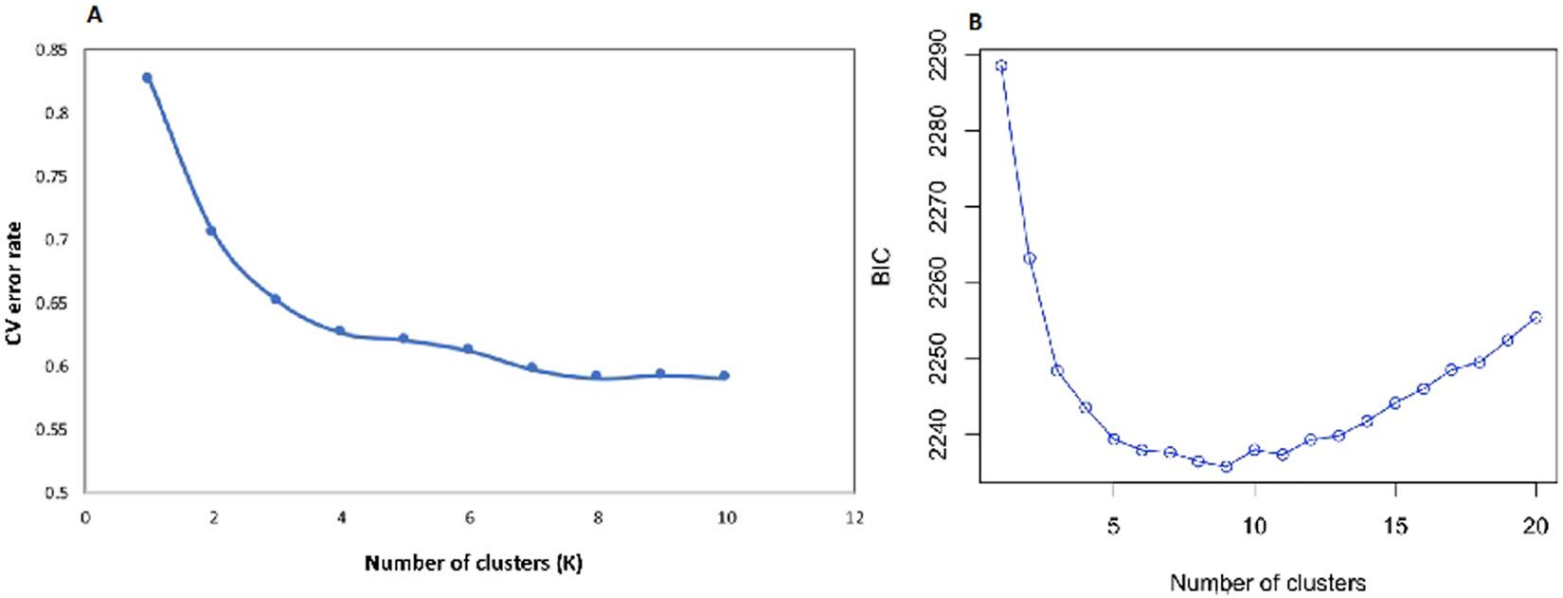
Graphs of (**A**) CV error against number of clusters K and (**B**) Bayesian Information Criterion versus number of clusters.

The three complementary methods used in determining the number of clusters among the sub-set of the cowpea mini-core population all show the presence of three major clusters. The error rate from cross-validation method used by ADMIXTURE and the Bayesian information criterion (BIC) from discriminant analysis of principal components (DAPC) to determine the appropriate number of sub-populations show rapid decline from K = 1 to K = 3 and from 1 to 3 respectively (Fig. 2), indicating that the samples can be grouped into three main clusters.

**Figure 3:**
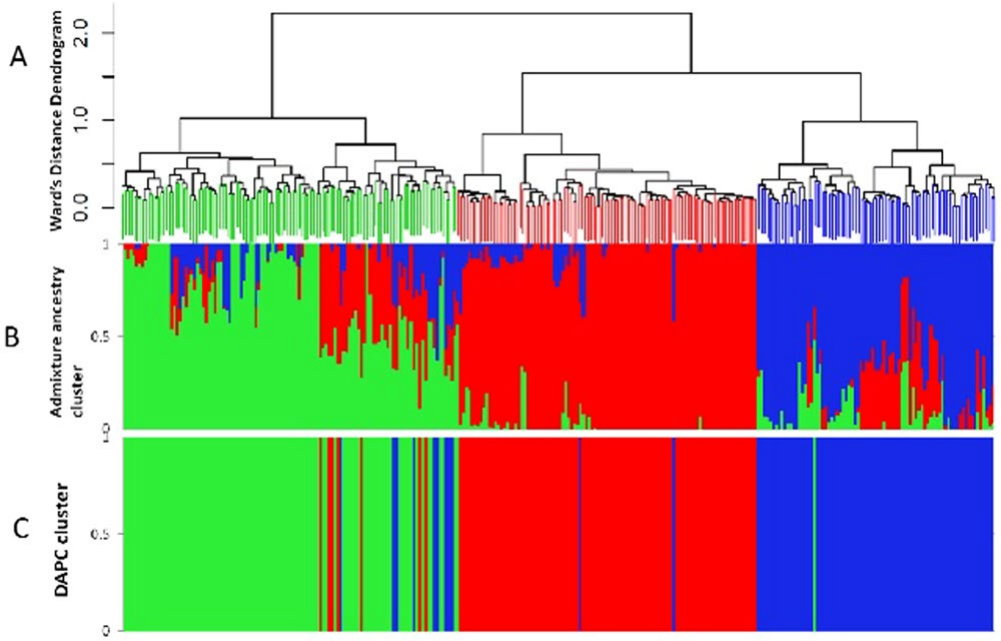
(**A**) Dendrogram of hierarchical clustering of 298 cowpea accessions; (**B**) Admixture ancestry Cluster; (**C**) DAPC cluster of 298 accessions based on 2,276 SNP markers (K = 3).

**Figure 4:**
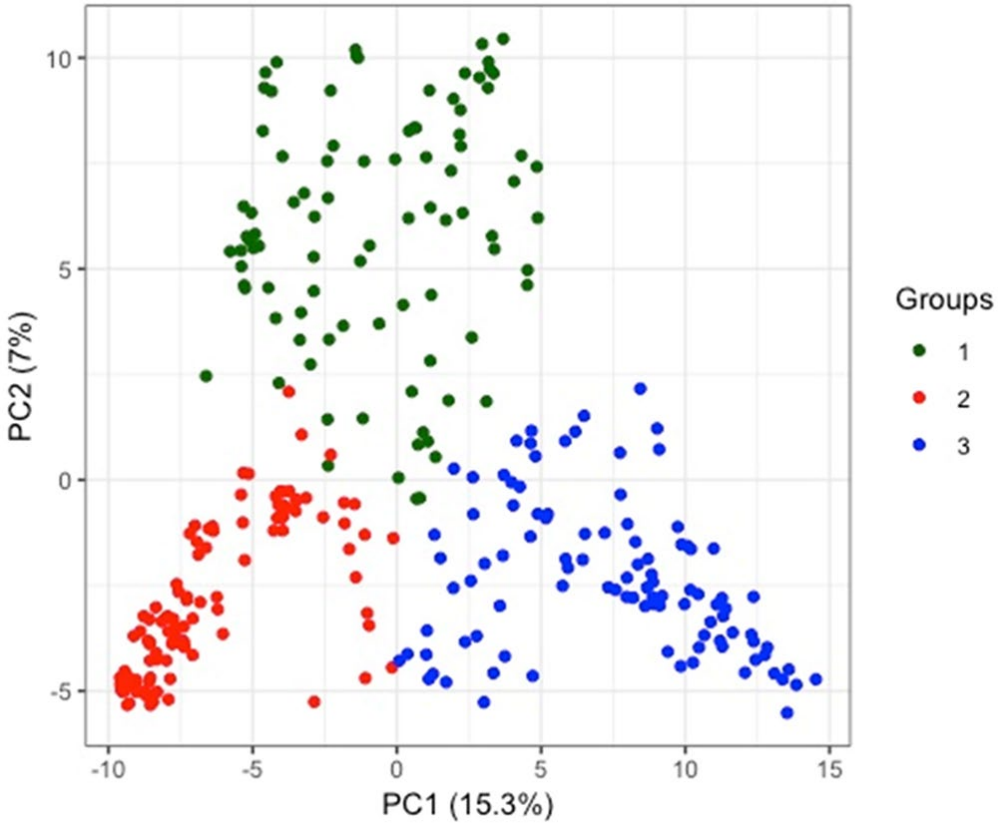
DAPC scatter plot of the 298 accessions along PC1 and PC2 axes.

The distribution of the tested cowpea mini core lines based on hierarchical clustering dendrogram (Fig. 3A), the model-based structure analysis (Fig. 3B), DAPC plot (Fig. 3C) and scatter plot from DAPC (Fig. 4) show that the accessions are divided into three major clusters. Groups of the DAPC plot and the arms of the dendrogram correspond to K = 3 of the admixture plot. Assignments of the 298 cowpea accessions into the three clusters identified on the Ward’s distance hierarchical dendrogram, DAPC and Admixture ancestry are generally in agreement. On the dendrogram, the number of lines per cluster varied from 81 (cluster 3) to 115 (cluster 1) (Supplementary Table 1). The 55 accessions from Nigeria are distributed into the three clusters with about half the number (28) in cluster one. Cluster two has 23 Nigerian accessions, while cluster three has four accessions. The 33 accessions from India are also distributed into the three clusters, 11 in cluster one, three in cluster two and 19 in cluster three. Most accessions (22) from Niger are in cluster two while the remaining two are in cluster three. There is no cluster made up exclusively of accessions from the same country although there are some cases where all accessions from same country belong in same cluster. For example, all four accessions from Lesotho are in cluster one and the four from Senegal are in cluster two. Ninety-two of the 102 lines (approx. 90%) in cluster two are from 13 West and Central African countries while the others are from Tanzania, Botswana, India, USA and two from unknown countries. Cluster three with the lowest number of accessions contained all the lines from Egypt (14) except one that is in cluster one as well as most of the accessions from East and Southern Africa.

Membership clustering for DAPC ranged from 91 to 107. Group one is made up of 100 accessions with most accessions from Nigeria. Group two has 107 accessions with majority of them from Nigeria and Niger while group three has 91 accessions with most of the accessions coming from India and Egypt. Group membership from DAPC is to a large extent in agreement with the hierarchical dendrogram clustering. Group one has 99 accessions in agreement with the hierarchical dendrogram out of the 100 members assigned by the DAPC while group two has 100 accessions in agreement with hierarchical dendrogram out of the 107 members assigned to the group. The third group with 91 accessions has 81 accessions in agreement with the hierarchical dendrogram.

Principal component analysis (PCA) was performed and it further supported the groupings of the samples based on the membership assignment from DAPC (Fig. 4). The first principal component explains 15.3 percent of variation and the second explains seven percent. Both explain 22.3% of the total variation (Supplementary Table 2).

### Genetic diversity and population differentiation of observed groups

Diversity indices statistics for the three observed groups show that, irrespective of clustering method used, group one shows the highest polymorphic information content, number of effective alleles, expected and observed heterozygosity (Table 2). However, depending on the clustering method that was used the values vary for groups two and three.

**Table 2:** Diversity indices statistics for the different observed groups.

Category	No of cluster analysis	PIC	Ne	He	Ho
Groups based on accessions					
1	115	0.241	1.497(0.004)	0.298(0.003)	0.089
2	102	0.157	1.314(0.007)	0.192(0.004)	0.068
3	81	0.209	1.424(0.007)	0.257(0.004)	0.066
Groups based on DAPC					
1	100	0.249	1.499 (0.006)	0.306 (0.003)	0.081
2	107	0.245	1.484 (0.006)	0.299(0.003)	0.073
3	91	0.245	1.472 (0.007)	0.290 (0.003)	0.073

PIC = polymorphic information content; Ne = number of effective alleles; He = expected heterozygosity; Ho = observed heterozygosity.

Analysis of molecular variance was also carried out on the identified groups on the hierarchical dendrogram and DAPC. The results of analysis are identical irrespective of the clustering method used for the structure analysis. The observed variance partitioned among the three groups is 16 percent, 66 percent of the variance partitioned among individual accessions and 18 percent within the accessions (Table 3).

**Table 3:** Analysis of molecular variance for variation among and within sub population based on 2,276 SNPs and 298 cowpea accessions.

Source	DF	SS	MS	% Est. Var.	F-Statistics		P
Groups based on hierarchical Dendrogram							
Among observed clusters	2	29313.3	14656.7	16	F_ST_	0.16	0.001
Among accessions	295	196113.9	664.8	66	F_IS_	0.78	0.001
Within accessions	298	24253.5	81.4	18	F_IT_	0.82	0.001
Total	595	249680.7		100	F’_ST_	0.24	
Groups based on DAPC							
Among observed clusters	2	29435.5	14717.8	16	F_ST_	0.16	0.001
Among accessions	295	200419.3	679.4	66	F_IS_	0.79	0.001
Within accessions’	298	24253.5	81.4	18	F_IT_	0.82	0.001
Total	595	254108.3		100	F’_ST_	0.24	

There is a moderate amount of differentiation between the three groups (F_ST_ = 0.16, F’_ST_ = 0.24), indicating that the groups are relatively genetically distinct. The F_IS_ and F_IT_ values are 0.78 and 0.82 respectively indicating that the cowpea lines making up the groups are inbred lines.

## Discussion

The world’s largest cowpea germplasm collection maintained at the International Institute of Tropical Agriculture (IITA) headquarters in Ibadan Nigeria has more than 15,000 landraces and over 2,000 wild relatives. These accessions have served and continue to serve as sources of genes for desirable traits contributing to the successes being recorded in variety development especially in sub-Saharan Africa. In this study involving a sub-set of the mini core collection, a low number of robust SNP markers was observed which can be attributed to the non-availability of reference SNP discovery pipeline (when the study was performed) or the acknowledged narrow genetic base of cowpea^13,28^. The observed genetic distance based on markers between pairs of randomly selected members of the cowpea mini core range between 0.0096 and 0.462 (Fig. 1) while in an earlier study by Huynh *et al*.^5^ genetic distances based on shared alleles among a collection of cowpea ranged from 0.01 to 0.72. Following the use of gene-derived markers and sequencing on the USDA *Vigna* germplasm collection it was concluded that genetic diversity present in cowpea was minimal and genetic distances among accessions low^12^. Vaillancourt and Weeden^29^ had also earlier reported very low level of chloroplast DNA diversity in landraces compared to wild cowpea and based on the observations concluded that 1) the domesticated form was derived from a narrow selection of the wild germplasm and 2) chloroplast gene flow between wild and cultivated types has been very limited. In addition, the authors detected no homoplasy in tested cultivated and wild relatives following construction of a hierarchical tree. Genetic diversity was reported to be low among cowpea populations collected from Benin Republic^30^, Ghana^31^ and Sudan^32^. In explaining what could be responsible for the narrow genetic base for cowpea Coulibaly *et al*.^1^ suggested that a single domestication event may have occurred between cultivated and the wild progenitor and this concurs with one of the conclusions reached by Vaillancourt and Weeden^29^.

The relatively high F_IS_ mean value of 0.75 obtained across samples indicated that most accessions are inbred. Xiong *et al*.^7^ also reported low heterozygosity among the 768 cowpea accessions used in their diversity study. These observations are to be expected since cowpea is a highly self-pollinated crop. Depending on the genotype and environment the extent of outcrossing in cowpea is low and could range from less than 0.15 to up to 1.58%^33^. However, He = 0.4344 in a population comprising 105 cowpea accessions from Kenya, Niger, Nigeria and China has recently been reported^34^.

The sampled mini core accessions of the world cowpea collection maintained at IITA and used in the present study are distributed into three main clusters on the dendrogram generated following hierarchical cluster analysis. These three clusters are also identified by DAPC and ADMIXTURE. The distribution of the germplasm accessions into three main clusters in this study agrees with the findings by Xiong *et al*.^7^ that reported a diverse set of 768 cowpea germplasm maintained at United States Department of Agriculture – Germplasm Resources Information Network (USDA-GRIN) formed three well defined groups and Qin *et al*.^35^ who also found three clusters among 369 accessions belonging to the USDA’s core collection. It can therefore be stated, based on the results of the present study, that the set of mini core lines we have used represent a significant amount of the diversity present in the world cowpea collection maintained at IITA. Hence, the test lines represent a broad picture of the crop’s genetic diversity. It is also worth noting that the 369 USDA cowpea core collections came from 47 countries whereas the 298 mini core accessions we used are obtained from 50 countries.

Two of the three groups formed in this study have most of their members collected from different parts of Africa thus agreeing with results by Chen *et al*.^34^ who found that two of the four clusters from their study were made up of accessions from Niger and Nigeria and the other made of accessions from Kenya. The identification of three clusters (gene pools) each from America (North America and Latin America), the three regions of Africa (West and Central Africa, East and Southern Africa) and Central West Asia, Europe and Oceania were reported^7^. The composition of the three clusters identified in our study does not agree with these observations when sources of their origin are considered. Rather, our results align more with those of Huhyn *et al*.^5^ in their study in which they identified three gene pools among 422 accessions that included 46 wild cowpea relatives.

The presence of accessions from West and Central Africa especially Nigeria in each of the three groups is further evidence that the sub-region is a centre of diversity for cultivated cowpea^36^. Many authors have submitted that the center of diversity for cultivated cowpea is the West and Central African sub-region^2,37^ from where it moved to the other parts of the world - Asia, North and South America and Europe^5,38^.

The germplasm lines making up the bulk of IITA’s cowpea mini core did not cluster according to countries of origin despite that geographical location along with agronomic and botanical descriptors were used to define the core collection^4^. However, many of the accessions from West and Central Africa constitute the majority of members in group two while most members of group three are from East and Southern Africa. Clustering of accessions according to countries of origin varies across crop species. For example, in African rice (*Oryza glabberima*), a self-pollinated crop like cowpea clustering of genotypes was more according to country of origin than to agro-ecology^39^ whereas in lentil only a weak correlation was observed between geographic origin and genetic relationships among tested landraces^40^. Cowpea lines from Niger and Nigeria clustered together and separated from those collected from Kenya and China^34^. Clustering together of genotypes from Niger and Nigeria was attributed to their coming from similar agro-ecologies^34^. In their study Xiong *et al*.^7^ reported no clustering of cowpea genotypes according to country of origin. However, geographic variation in germplasm distribution is nearly always impossible to separate from ecologically determined variation hence the recognition of ‘ecogeographic factors’ in plants’ genetic diversity^41^. It is reasonable to believe that the cowpea germplasm lines have remained in West and Central Africa sub-region over a long period after having been domesticated there. The plants have become adapted to the agro-ecologies prevalent in the sub-region rather than countries especially since a common farming system of intercropping is mostly practised in the different countries. Also, the high level of self-pollination, as in cowpea, may have resulted in the rapid fixation of alleles and accumulation of mutant genes in tomato^42^. It is also worth noting that movement of seeds among farmers in the West and Central Africa sub-region is not limited by national boundaries as farmers in neighbouring communities exchange seeds freely with one another^39^ and farmer-to-farmer seed movement has been recognised as a means of disseminating seeds of newly improved varieties.

The genetic diversity within cluster two of which 92 percent of members are from West and Central Africa is lowest of the three clusters. Total genetic variation among cowpea accessions from outside of Africa was slightly greater than in the African landraces^5,34,35^. Low levels of precipitation in the areas of sub-Saharan Africa (SSA) where cowpea is mostly found may partly contribute to the relatively lower genetic diversity in lines from the region as compared to those from other parts of the world where more precipitation occurs. For example, an appreciable level of genetic diversity has been reported among yard-long-bean (*V. unguiculata* ssp. *sesquipedalis*) found mostly in the moist Asian countries especially China and India^43^. The yard-long-bean evolved in Asia from the cultivated cowpea introduced originally from the savannahs of SSA where they are not found. The greater precipitation and lower sunlight in Asia was suggested as being responsible for the evolution of the yard-long-bean from cowpea^5^. A relatively higher genetic diversity has been reported in tomatoes from the western Galapagos islands and this was attributed to the higher precipitation in the area^44^. It has also been suggested that plant species richness is correlated with annual precipitation as observed in the Neotropics^45^.

In this study, members of the cluster with the most number of accessions (115 accessions) are from 27 countries 16 of which are from outside of Africa and 11 African countries. This also showed the highest level of genetic variation among the three clusters. The higher genetic variation observed among accessions in this most cosmopolitan cluster may be the result of the high number of countries from where the members are collected. Most genetic variance in the USDA cowpea world collection was found within, instead of, among geographic regions and within instead of among countries^7^.

The analysis of molecular variance (AMOVA) showed that highest variation was present among accessions followed by within accessions and smallest among clusters. Similarly, the highest amount of variation was found among individuals, followed by within individuals and least among populations^34^. There is therefore, in cowpea higher genetic divergence among individual accessions which could not be explained by the groups. However, in African rice molecular variance was reported to be highest within than among groups when based on structure and cluster analysis and the diversity could not be explained by ecology^39^.

Accessions from the USA were distributed among different clusters but the most number were found in cluster one where they are found to be closely associated with accessions from West Africa thus suggesting that relatively high similarities exist between USA and West African lines. The observed 86% similarity between American breeding lines and accessions from West Africa agree with this observation^7^. It can be suggested that many parental lines used for making crosses in the USA while generating breeding lines may be of West African origin. Whit^46^ suggested that cowpea came to the USA through slaves who may have brought them along from West Africa. We also observed that some USA lines are in close proximity with accessions from Egypt and India. This may justify the findings that some of the USA lines came through other sources than from only West Africa^5^. It had been suggested that cowpea germplasm was also dispersed from sub-Saharan Africa to Europe and elsewhere through Egypt in North Africa^47^.

The bulk of cowpea accessions maintained in the Genetic Resources Center at IITA (>71%) are from Africa and would therefore be mostly landraces. These lines would to a very large extent represent the diversity present in the indigenous cultivated cowpea. The history of cowpea variety development in Africa is recent. Early reports on genetic studies in cowpea are from the USA and date back to early 1900s. Inheritance of several seed related traits in cowpea were the earliest reported studies from the USA^48,49^. Available records show that the work carried out at the Agricultural Research Station, Bihar, India in which crosses were made to generate segregating populations from where early maturing lines with economically desirable traits were selected may probably be one of the earliest in the history of cowpea variety development^50^. Although most cowpea is produced and consumed in Nigeria, only limited improvement activities were carried out in the country until 1960s. Widespread dissemination of improved varieties to farmers in the West Africa sub-region is even more recent and can be traced to the establishment in early 1980s of the Semi-Arid Food Grain Research and Development (SAFGRAD) Program with support from the USAID.

This diversity study was carried out using genotyping by sequencing on a sub-set of the mini core from the world’s largest assemblage of cowpea germplasm collections and the results further confirm West and Central Africa as center of origin for cowpea. Further, the constitution of the mini core collections was successful in encompassing the diversity that may be present in the cowpea gene bank maintained at IITA as three groups were identified which is in concurrence with previous diversity studies in cowpea. It can be inferred from the study therefore that SNP markers are effective in placing cowpea germplasm lines in appropriate clusters based on relationships at molecular level and with the current accumulation of genomic tools in cowpea the crop promises to benefit from the deployment of these tools especially in the development of new varieties that should meet the immediate and future challenges facing its productivity.

## Methods

### Plant materials and DNA extraction

Seeds of the 370 accessions making up the cowpea mini-core collections were obtained from the IITA’s Genetic Resources Center, sown in pots containing 5.0 kg top soil and placed in the screenhouse. DNA was extracted from a newly expanded young trifoliate leaf of emerged seedlings of each line using SDS method^51^ with some modifications. DNA concentrations of samples were normalized and working solutions contained between 10–100 ng/µl. Ninety-five samples were placed in a 96 well plate. As a DNA quality measure, 5 µl of each of 10 samples in a plate were digested using *Eco*RI restriction enzyme and run on a 1% w/v agarose gel along with the λ *Hind*III size standards.

### Genotyping-by-sequencing (GBS)

Genome reduction for sequencing was achieved by digesting genomic DNA with the restriction enzyme, ApeKI, which recognizes a degenerate 5 bp sequence (GCWGC, where W can be A or T), and sequencing was performed following the standard procedure^14^. A total of about four 96-plex GBS libraries were constructed and sequenced on the Illumina HiSeq 2000. Due to unavailability of reference genome (as at the time of the sequencing), SNP variants were discovered using the UNEAK pipeline^18^ (http://www.maizegenetics.net/gbs-bioinformatics) as implemented in TASSEL Version: 5.0^52^ (Version: 3.0.166 Date: April 17, 2014). In summary, reads were trimmed to a 64-bp length and unreliable markers filtered out using a network filter, designed to detect and eliminate markers showing unexpected relationships with others that could be a result of paralogous sequences and/or sequencing errors^18^. A total of 121,910 SNPs was called out of the UNEAK pipeline. The called SNPs were further filtered using TASSEL software^52^. SNPs with more than 20% missing data were removed as were those with minor allele frequency (MAF) below 0.05. Non-polymorphic markers i.e., having a variance close to 0 were identified (only six markers were discarded based on this criterion). SNPs resulting from sequence paralogy were further removed, resulting in a final set of 2,276 SNPs.

### Data analysis

#### Genetic diversity, population structure and phylogenetic analyses

Diversity analysis was carried out using the filtered 2,276 SNPs. Genetic diversity parameters which include minor allele frequency (MAF), polymorphic information content (PIC), the effective number of alleles (Ne), expected heterozygosity (He), observed heterozygosity (Ho) and inbreeding coefficient (Fis) were established using GenAlex version 6.41^53^, power marker^54^ and PLINK^55^.

Population structure was characterized using three complementary approaches: 1) distance-based hierarchical clustering analysis; 2) a model-based maximum likelihood estimation of ancestral sub-populations using ADMIXTURE^56^ and 3) assumption-free discriminant analysis of principal components (DAPC)^57^. For the hierarchical clustering, the pairwise genetic distance (identity-by-state, IBS) matrix was calculated among all individuals using PLINK^55^. A Ward’s minimum variance hierarchical cluster dendrogram was then built from the IBS matrix using the Analyses of Phylogenetics and Evolution (ape) package^58^ implemented in R^59^.

In the second approach, population structure and accession ancestry were determined using the ADMIXTURE method which assumes linkage equilibrium among loci and Hardy-Weinberg equilibrium within ancestral populations^56^. For the analysis, the number of subpopulations, K, varied from 2 to 10. According to ADMIXTURE’s cross-validation procedure a good value of K will exhibit a low cross-validation error compared to other K values^55^. Hence, the most appropriate K value that is useful and better describes the data and also has good correspondence with the clustering pattern obtained by the hierarchical tree, was selected after considering the 10-fold cross-validations.

To complement the results from ADMIXTURE, we carried out DAPC using the R package ‘adegenet’. The optimal number of clusters from DAPC was inferred using k-means analysis by varying possible number of clusters from two to 40 using a Bayesian Information Criterion (BIC) to assess the best supported model. The number of clusters at which BIC decreases were considered to determine the most likely K value. Following the information based on hierarchical clustering and ADMIXTURE analysis the most appropriate K expected to provide useful summaries of the data was selected. DAPC clustering was thereafter performed on the clusters identified using the first 70 principal components. The membership probabilities of each individual for the different groups were obtained from DAPC and the results of DAPC analysis, ADMIXTURE, and the hierarchical tree were compared. As sub-clusters were observed within the mini core samples, the fixation index (F_ST_) and standardized F_ST_ (F’_ST_,) of the observed sub-clusters were assessed using analysis of molecular variance (AMOVA) implemented in GenAlex 6.41 and the genetic diversity parameters of each observed cluster determined. Genetic diversity parameters were also calculated for each of the identified sub-clusters.

## Electronic supplementary material

Supppementary Table 1

Supplementary Table 2

## Data Availability

The data analysed in this study were sent along with the submitted manuscript (Supplemental Information).

## References

[1] Coulibaly S, Pasquet RS, Papa R, Gepts P (2002). AFLP analysis of the phenetic organization and genetic diversity of *Vigna unguiculata* L. Walp. reveals extensive gene flow between wild and domesticated types. Theor. Appl. Genet..

[2] Padulosi, S., & Ng, Q. Origin, taxonomy and morphology of *Vigna unguiculata* (L.) Walp., In *Advances in Cowpea Research*, eds Singh, B. B., Mohan Raj, D. R., Dashiell, K. E. & Jackai, L. E. N. Co-publication of International Institute of Tropical Agriculture (IITA) and Japan International Research Center for Agricultural Sciences (JIRCAS). IITA, Ibadan, Nigeria pp. 1–12 (1997).

[3] Ng, Q. & Maréchal, R. Cowpea taxonomy, origin and germplasm, in *Cowpea Research, Production and Utilization*, eds Singh, S. R. & Rachie K. O. John Wiley and Sons, Chichester. pp 11–21 (1985).

[4] Mahalakshmi V, Ng Q, Lawson M, Ortiz R (2007). Cowpea [*Vigna unguiculata* (L.) Walp.] core collection defined by geographical, agronomical and botanical descriptors. Plant Genet. Res..

[5] Huynh Bao-Lam, Close Timothy J., Roberts Philip A., Hu Zhiqiu, Wanamaker Steve, Lucas Mitchell R., Chiulele Rogerio, Cissé Ndiaga, David Antonio, Hearne Sarah, Fatokun Christian, Diop Ndeye N., Ehlers Jeffrey D. (2013). Gene Pools and the Genetic Architecture of Domesticated Cowpea. The Plant Genome.

[6] Padulosi, S. Genetic diversity, taxonomy and ecogeographic survey of the wild relatives of cowpea (*Vigna unguiculata*). PhD thesis. University of Louvain La Neuve, Belgium (1993).

[7] Xiong H (2016). Genetic diversity and population structure of cowpea (*Vigna unguiculata* L. Walp.). PLoS ONE.

[8] Frankel, O. H. & Brown A. H. D. Plant genetic resources today: a critical appraisal, in *Crop Genetic Resources: Conservation and Evaluation*. eds Holden, J. H. W. & Williams J. T. London: George Allen & Unwin, pp. 249–257 (1984).

[9] Upadhyaya HD, Ortiz R (2001). A mini core collection for capturing diversity and promoting utilization of chickpea genetic resources in crop improvement. Theor. Appl. Genet..

[10] Upadhyaya, H. D., Gowda, C. L. L., & Sastry, D. V. S. Management of germplasm collections and enhancing their use by mini core and molecular approaches. APEC-ATCWG Workshop. Capacity Building for Risk Management Systems on Genetic Resources. p. 35–70 (2008).

[11] Fatokun CA, Danesh D, Young ND, Stewart EL (1993). Molecular taxonomic relationships in the genus *Vigna* based on RFLP analysis. Theor. Appl. Genet..

[12] Wang ML, Barkley NA, Gillaspie GA, Pederson GA (2008). Phylogenetic relationships and genetic diversity of the USDA Vigna germplasm collection revealed by gene-derived markers and sequencing. Genet. Res..

[13] Li C-D, Fatokun CA, Ubi B, Singh BB, Scoles GJ (2001). Determining genetic similarities and relationships among cowpea breeding lines and cultivars by microsatellite markers. Crop Sci..

[14] Elshire RJ (2011). A robust, simple genotyping-by-sequencing (GBS) approach for high diversity species. PLoS ONE.

[15] Deschamps S, Llaca V, May GD (2012). Genotyping-by-Sequencing in Plants. Biology.

[16] Poland JA, Rife TW (2012). Genotyping-by-sequencing for plant breeding and genetics. Plant Genome.

[17] Girma G (2014). Next-generation sequencing based genotyping, cytometry and phenotyping for understanding diversity and evolution of guinea yams. Theor. Appl. Genet..

[18] Lu F (2013). Switchgrass genomic diversity, ploidy, and evolution: Novel insights from a network-based SNP discovery protocol. PLoS Genetics..

[19] Poland JA, Brown PJ, Sorrells ME, Jannink J-L (2012). Development of high-density genetic maps for barley and wheat using a novel two-enzyme genotyping-by-sequencing approach. PLoS ONE.

[20] Morris GP (2013). Population genomic and genome-wide association studies of agro-climatic traits in sorghum. Proc. Natl. Acad. Sci. USA.

[21] Poland J (2012). Genomic selection in wheat breeding using genotyping-by-sequencing. Plant Genome.

[22] Singh BP, Singh B, Mishra S, Kumar V, Singh NK (2016). Genetic diversity and population structure in Indian wild rice accessions. Austral. J. Crop Sci..

[23] Emanuelli F (2013). Genetic diversity and population structure assessed by SSR and SNP markers in a large germplasm collection of grapes. BMC Plant Biol..

[24] Sonah, H. *et al*. An improved genotyping by sequencing (GBS) approach offering increased versatility and efficiency of SNP discovery and genotyping. *PLoS ONE* **8**(1), e54603. doi:10.137 (2013). 10.1371/journal.pone.0054603 PMC3553054 23372741

[25] Raybould AF, Mogg RJ, Clarke RT (1996). The genetic structure of *Beta vulgaris* ssp. *maritima* (sea beet) populations: RFLPs and isozymes show different patterns of gene flow. Heredity.

[26] Perez de la Vega M, Garcia P (1997). Genetic structure of self-pollinating species: the case of wild. Avena. Bocconnea.

[27] Allard RW (1988). Genetic changes associated with the evolution of adaptedness in cultivated plants and their wild progenitors. J. Heredity.

[28] Asare A (2010). Assessment of the genetic diversity in cowpea (*Vigna unguiculata* L. Walp.) germplasm from Ghana using simple sequence repeat markers. Plant Genet. Res..

[29] Vaillancourt RE, Weeden NF (1992). Chloroplast DNA polymorphism suggests Nigerian center of domestication for the cowpea, *Vigna unguiculata* (Leguminosae). Am. J. Bot..

[30] Zannou A (2008). Genetic variability of cultivated cowpea in Benin assessed by random amplified polymorphic DNA. Afric. J. Biotech..

[31] Egbadzor KF, Danquah EY, Ofori K, Yeboah M, Offei SK (2014). Diversity in 118 cowpea (*Vigna unguiculata* (L.) Walp.) accessions assessed with 16 morphological traits. Inter. J. Plant Breed. Genet..

[32] Ali ZB (2015). Assessing the genetic diversity of cowpea (*Vigna unguiculata* (L.) Walp.) accessions from Sudan using simple sequence repeat (SSR)markers. . Afric. J. Plant Sci..

[33] Fatokun CA, Ng Q (2007). Outcrossing in cowpea. J. Food, Agric. Environ..

[34] Chen H (2017). Genetic diversity and a population structure analysis of accessions in the Chinese cowpea [*Vigna unguiculata* (L.) Walp.] germplasm collection. The Crop J..

[35] Qin J (2016). Population structure analysis and association mapping of seed antioxidant content in USDA cowpea (*Vigna unguiculata* L. Walp.) core collection using SNPs. Canad. J. Plant Sci..

[36] Steele M (1976). Cowpea, *Vigna unguiculata* (Leguminosae-Papillionatae), in *Evolution of* Crop Plants, ed Simmonds, N. Longman, London U.K. Theor. Appl. Genet..

[37] Faris DG (1965). The origin and evolution of the cultivated forms of *Vigna sinensis*. Canad. J. Genet. Cytol..

[38] Steel, W. M. & Mehra, K. L. Structure, evolution and adaptation to farming systems and environments in *Vigna*, in *Advances in Legume Science*, ed. Summerfield, R. J. & Bunting, A. H. Kew Royal Gardens. pp 393–404 (1980).

[39] Ndjiondjop, M-N. *et al*. Genetic variation and population structure of *Oryza glabberima* and development of a mini core collection using DArTSeq. *Front. Plant Sci*. 10.3389/fpls.2017.01748 (2017). 10.3389/fpls.2017.01748 PMC5651524 29093721

[40] Lombardi M (2014). Assessment of genetic variation within a global collection of lentil (*Lens culinaris* Medik.) cultivars and landraces using SNP markers. BMC Genetics.

[41] Rao VR, Hodgkin T (2002). Genetic diversity and conservation and utilization of plant genetic resources. Plant Cell Tiss. Organ Cult..

[42] Rick, C. M. Genetic variation and evolution in Galapagos tomatoes, in *Patterns of Evolution in* Galapagos *Organisms*, eds. Bowman, R. I. Berson, M. & Leviton, A. (San Francisco, CA: *American Association for the Advancement of Science*) 97–106 (1983).

[43] Xu P (2017). Genomic regions, cellular components and gene regulatory basis underlying pod length variations in cowpea (*V. unguiculata* L. Walp.). Plant Biotechnol. J..

[44] Rick CM, Fobes JF (1975). Allozymes of Galapagos tomatoes: polymorphism, geographic distribution, and affinities. Evolution (N. Y.).

[45] Gentry AH (1982). Patterns of neotropical plant species diversity. Evol. Biol..

[46] Whit, W. C. Soul food as cultural creation, in *African American foodways: Explorations of history and culture*, ed. Bowser, A. University of Illinois Press, Urbana, IL. p. 45–58 (2007).

[47] Ng, Q. & Singh, B. B. “Cowpea,” in *Biodiversity in Trust. Conservation and use of plant genetic resources in* CGIAR Centres, eds Fuccillo, D., Sears, L. & Stapleton, P. Cambridge University Press. Pp 82–99 (1997).

[48] Spillman WJ (1911). Inheritance of the ‘eye’ in Vigna. Am. Natural..

[49] Spillman WJ (1913). Color correlation in cowpea. Science.

[50] Roy RS, Richharia RH (1948). Breeding and inheritance studies on cowpea, *Vigna sinensis*. J. Am. Soc. Agron..

[51] Dellaporta SL, Wood J, Hicks JB (1983). A plant DNA minipreparation Version II. Plant Mol. Biol. Rept..

[52] Bradbury PJ (2007). TASSEL: software for association mapping of complex traits in diverse samples. Bioinformatics.

[53] Peakall R, Smouse PE (2006). GENALEX 6: Genetic Analysis in Excel. Population Genetic Software for Teaching and Research. Molecular Ecology Notes.

[54] Liu K, Muse SV (2005). PowerMarker: Integrated Analysis Environment for Genetic Marker Data. Bioinformatics.

[55] Alexander DH, Novembre J, Lange K (2009). Fast model-based estimation of ancestry in unrelated individuals. Genome Res..

[56] Jombart T, Devillard S, Balloux F (2010). Discriminant analysis of principal components: A new method for the analysis of genetically structured populations. BMC Genetics.

[57] Purcell S (2007). PLINK: a tool set for whole-genome association and population-based linkage analyses. Am. J. Human Genet..

[58] Paradis E, Claude J, Strimmer K (2004). APE: Analyses of phylogenetics and evolution in R language. Bioinformatics.

[59] R Core Team. R: A language and environment for statistical computing. R Foundation for Statistical Computing Vienna, Austria (2013).

